# Supplementary material for: Augmented reality in healthcare education: an integrative review
Source: PeerJ. 2014 Jul 8;2:e469. doi: 10.7717/peerj.469 (PMC4103088; doi:10.7717/peerj.469)
Supplement: Appendix SI [file peerj-02-469-s001.doc]

*Appendix I Description of 25 comparative studies included in the integrative review of AR in medical education*

| Study | Research (Quantitative) | | | | AR Technology | | | | Learning | | |
| --- | --- | --- | --- | --- | --- | --- | --- | --- | --- | --- | --- |
|  | Aim | Design | Participant | Result | Display | Tracking | Input Devices | Develop tool | Theory | Strategy | Effect |
| (Rasimah Che, 2011) | Investigated user’s perception and acceptance of MR | Development, a pilot test, 22-item structured survey | 63 SBS (2^nd^ to 4^th^ year) | Willing to try. ITU MR is affected mostly by PU, moderately by PEOU and weakly by PE. PEOU has strong positive correlation with PU and influence on PE, moderately with PI. | Monitor- based | Web camera two-dimensional fiducially mark | Web camera, keyboard | FLARToolkit 3D tools, Photoshop, Sound Forge | Situated learning | Demonstration Brief hands on Task-oriented | Not clear because it has not been tested. |
| (Yeo et al., 2011) | Determined if AR guidance systems can assist medical trainees in learning essential skill | Development, randomized experiment, 2 groups, post-test only | 40 = 26MS +9BES +5R 1^st^ year ) | The Overlay group performed better with less tissue trauma than the Control group. The system helped not only to avoid the initial period of high errors and lengthy procedures, but also improved overall accuracy and efficiency. | External monitor, semi-reflective glass | Electromagnetic tracker, radiographic markers | Perk Station phantom control box, a sensor attached to a needle | 3D Slicer XML | None | Image and laser guidance compare with classical freehand method | Decreased the amount of practice required to become eligible for clinical procedures, |
| (Luciano et al., 2011a) | Evaluated the learning retention on a augmented reality and haptic technology workstation | Experiment one group, post-test only | 51 fellows and R | The reduced failure rate from 16.9% to 12.5% Furthermore, The performance accuracy showed a 15% mean score improvement and more than a 50% reduction in standard deviation from practice to test. | High- resolution stereosco-pic display | An electromagnetic head-and-hand tracking system landmarks | the haptic stylus an electromagnetic sensor. | The Immersive-Touch system (the system name) | None | Practice guided by fluoroscopic images | Reduced failure rate and improved performance accuracy indicating positive learning. |
| (Feifer, Delisle, & Anidjar, 2008) | Examined the usefulness, reliability and applicability of the smoothness metric of the ProMIS hybrid simulator | Nonrandom experiment two groups, test, post-test | 15R = 8PGYs (1 to 3) +7PGYs ( 4, 5) | Statistically significant differences in all MISTELS tasks were evident for all 6 sessions. The differentiating capabilities of the hybrid simulator were maintained even after additional teaching in the junior group. | Computer screen (?) | Camera tracking systems marked electrical tape | Camera, real surgical instruments | The ProMIS (the system name) | None | Unsupervised simulator practice | A valuable asset for preparatory training for live operative experience, allowing improved trainee assessment. |
| (Ritter, Kindelan, Michael, Pimentel, & Bowyer, 2007) | To prove the ProMIS metrics could differentiate groups as well as standard FLS scoring with fewer personnel requirements | Non-random experiment, three groups test and re-test | 60 = 8AS /CR +44MS 3^rd^ year /I +8R (PGYs 2–4) | The more experienced the more outperformed. Statistically significant differences between the groups across all trials. A strong relationship between the FLS scores and the ProMIS metrics was apparent for three groups. | Computer screen? | Separate camera tracking systems marked | Actual laparoscopic surgical instruments plastic body mold with a neoprene cover | FLS and the ProMIS (the AR name) | The manual skills of FLS | Demonstration performed up to five consecutive trials of the task | Validation potential of remote assessment and training. Also lends nicely to proficiency- based training curricula. |

*: SBS: students in biomedical science. MS: medical student. BES: biomedical engineering students. R: residents. PGYs: postgraduate years. AS: attending surgeons. CR: chief residents. I: interns.

*Appendix I Continued*

| Study | | Research ( quantitative) | | | | AR Technology | | | | Learning | | |
| --- | --- | --- | --- | --- | --- | --- | --- | --- | --- | --- | --- | --- |
|  |  | Aim | Design | Participant^*^ | Result | Display | Tracking | Input Devices | Develop tool | Theory | Strategy | Effect |
| (Kotranza, Lind, & Lok, 2012) | The enhancement of cognitive-psychomotor tasks within MREs with real-time visual feedback of learner task performance | | Development, nonrandom experiment, three groups pre-test in I post-tests in II | 69 = 41MS +3I +15PAS +5R +5C in study I 13 = 8(from Study I) +5CS in study II | Novices receiving real-time feedback performed equivalently or better than more experienced practitioners. Skills improvement in the MRE transfers to the real-world task of CBE of human patients through repeated practice of CBE in the MRE. | Notebook screen | The infrared webcam and the infrared marker | Sensors IR marker infrared-seeing webcam physical breast model | Unsupervised machine learning techniques, information- visualization | Expert- novice | In study I: demonstrating, performed in study II, performed | The efficacy of real-time feedback improving performance in complex real-world tasks. |
| (Oostema, Abdel, & Gould, 2008) | | Determine the computer-derived metrics for a hybrid simulator correlated with laparoscopic surgical skill | Experiment, one group performed recorded analysis | 24 MS (3^rd^ year) +19R (PGYs 1–5) +3AS | Statistically significant correlation between experience and performance for all three metrics. | Computer | Not clear see ProMIS | Not clear see ProMIS | ProMIS | None | Demonstration videos, repeat the tasks until the metrics for three valid repetitions | Facilitating learning at a time convenient for trainees without the presence of instructors. |
| (Yudkowsky et al., 2012b) | | To study the impact of simulator practice on simulated and live surgical performance | Development pilot testing, one group pre and post-tests survey questionnaire | 16R (PGYs 1-4 and up) | Both simulation-based and live procedure outcome measures showed significant improvement after practice, demonstrating that skills obtained on the simulator could be transferred to the surgical setting. | High- resolution high-pixel density stereoscopic display | A head-and-hand tracking system | Haptic stylus CT scans | Computed tomographic scan images? | None | Random presentation practice case one-by-one | Especially by novice residents, may accelerate learning and shorten the learning curve. |
| (Nischelwitzer, Lenz, Searle, & Holzinger, 2007) | | How to design and develop applications for educational purposes with the use of the ARToolkit | Development usability test experiment, two group, pre and post - questionnaire | 18 children between 7 and 13 years | Used the MIBB and acquired more knowledge than just read the text and the audio guides. The interaction with the virtual organs seems to improve understanding the functionality of the alimentary canal. | HMD? | Webcam markers | The interaction control pad | ARToolKit | None | Interactive story | The possible potential of AR in the area of learning. |
| (Jan, Noll, Behrends, & Albrecht, 2012) | | Learning environment powered by the ubiquitous availability of mobile phones | Randomly two group structured questionnaire | 10MS 3^rd^ year | Above-average values for hedonic quality and being highly attractive for users. The AR group answered questions more correctly than the textbook group. | Mobile phone screen | Marker camera | Camera, navigation controls icon | XML | None | Self- learning; group learning | Easier to capture learners attention. Possibility for significantly improving the learning process. |

*PAS: physician assistant student. C: clinicians. CS: clerkship students

Appendix I *Continued*

| Study | Research ( quantitative) | | | | AR Technology | | | | Learning | | |
| --- | --- | --- | --- | --- | --- | --- | --- | --- | --- | --- | --- |
|  | Aim | Design | Participant^*^ | Result | Display | Tracking | Input Devices | Develop tool | Theory | Strategy | Effect |
| (Pretto, Manssour, Lopes, Silva, & Pinho, 2009) | Up-date the traditional training environment for LS by introducing image and sound resources into the training manikins | Development pilot study validation tests post-test interview questionnaire | 13 final year MS for validation tests. 70 medical residency examination candidates | The facial expressions and the body injuries stimulate the trainees in the direction of a more autonomous evaluation of the patient, which is very important for emergency care. | Projector, a mask placed on the manikin face. audio connectors | No tracking | Camera waistcoat audio- connectors speakers | Tailor-made software, ARToolkit, Expression Toolkit, C++ | None | Autonomous observation proper medical care | The implemented features are significantly relevant for emergency care training. |
| (S.Botden, Buzink, Schijven, Jakimowicz, 2007, 2008; S. Botden, Hingh, & Jakimowicz, 2009a, 2009b) | To evaluate the training system value and validate the assessment method，the face validity，the suturing module of the ProMIS AR | Nonrandom experiment, three groups/ two groups based on clinical experience structured questionnaire | 90 = 27I +25SR +7R +30 Sur +1Spec 55 = 21SR +4R +29Sur +1Spec  24  18 | The ProMIS is regarded as more realistic and having better haptic feedback and as being more useful for training surgical residents. It is a valid tool for objectively assessing laparoscopic suturing skills. | Computer screen? | Camera tracking systems marked electrical tape | Torso-shaped mannequin camera real surgical instruments | The ProMIS (the AR name) | None | Demonstration performed task | Useful for training surgical residents. |
| (LeBlanc et al., 2010; Leblanc, Delaney, Ellis, et al., 2010; Leblanc, Delaney, Neary, et al., 2010; Leblanc, Senagore, et al., 2010) | To compare laparoscopic colorectal skills acquisition among HAL and SL on an AR simulator and human cadaver | Randomized experiment two groups preformed recorded analysis | 38 = 29PS +9JS (5 fellows and 4R) 29PS 34PS | Better performances with the hand-assisted approach. Technical skills scores and generic events score was considerably better on the simulator than on the cadaver. Overall satisfaction was better for the cadaver than simulator. | Computer screen? | Cameras incorporated into the body of the simulator(?) for HAL A 0O laparoscope for SL (?) | Real surgical instruments | The ProMIS 2.5 (the AR name) | None | Demonstration simulator | The ability for novice surgeons to perform several complete operative procedures. |
| (Sakellariou, Ward, Charissis, Chanock, & Anderson, 2009) | To depict meaningful information enhance the learning process in an augmented  reality environment | Development experiment, two groups, pre and post-assessment open and structured questionnaire | 12 medical trainees | The traditional method group with a mean improvement in scores of 16%, whilst the VR method group improvement of 25%. Positively phrased statements regarding the educational approach scored very highly in the VR group. | CrystalEyes shutter glasses | Tracked by the table -sensors | Haptic glove 3D spatialised speakers |  | Activity-based learning | Not clearly | Better understanding of the spatial interrelationships of the structural elements of the canal. |

*: PS: practicing surgeons. JS: junior surgeons. SR: surgical residents. Sur: surgeon. Spec: specialist

Appendix I *Continued*

| Study | Research ( qualitative) | | | | | AR Technology | | | | | Learning | | | |
| --- | --- | --- | --- | --- | --- | --- | --- | --- | --- | --- | --- | --- | --- | --- |
|  | Aim | Design | Participant | | Result | Display | | Tracking | Input Devices | Develop tool | Theory | Strategy | | Effect |
| (Nilsson & Johansson, 2008) | To see whether AR technology is socially accepted by the staff at the hospital | Case study interviewed, open questionnaire, videotaped observation | 8 = 4Exp +4Nov) in case 1. 12 professional (OR) nurses and surgeons in case 2 | | The overall result from both cases studies shows a system that the participants like rather than dislike. The participants would like to use AR instructions in their future professional life. | HMD with camera in case 1. HMD and earphones and micro- phone | | Marker tracking, camera | Number pad with buttons in case 1. Voice input in case 2. | ARToolKit ARToolKit Plus ARTag integrated set of software tools | None | Demonstration performed task | | The users are positive towards AR systems as a technology for instructions in terms of usefulness and social acceptance. |
| (Lamounier, Bucioli, Cardoso, Andrade, & Soares, 2010) | To provide a friendly and intuitive interface based on AR | System architecture development test for the system | 2 volunteers | | More natural and intuitive interface different input types: the ECG image capture and the sensor, text input and load from a file confirmed the suitability of AR for health applications. | Computer screen | | Camera monochromatic marker | Sensor, Camera Keyboard | Simulation software. Irfraview Chromakey | None | Not clear | | It is useful and interesting tool that can help medical students. |
| (Davis et al., 2002; Rolland, 2003) | To propose an interactive tool for training that involves programming or instructor feedback | Development preliminary tests local and remote tests observation | no | | The average delays are the same for the local and remote tests, which proves that the application can be run across distributed platforms. | HMPD | | Polaris hybrid optical tracker | Not clear | METI. VESS Visible Human data sets. 3D CAD-model C/C++. | None | Not clear | | The system will allow paramedics to practice their skills and provide them with the visual feedback they could not otherwise obtain. |
| Study | Research ( quantitative, qualitative) | | | | | AR Technology | | | | | Learning | | | |
|  | Aim | Design | | Participant | Result | Display | Tracking | | Input Devices | Develop tool | Theory | | Strategy | Effect |
| (Rosenbaum, Klopfer, & Perry, 2007) | Do students understand the dynamic nature underlying the game and perceive it as an authentic experience? | Phenomenology experiment, 3 groups pre and post-survey, interviews video-taped and transcribed. | | 21 high school students (15 girls and 6 boys) | Students perceive the game as authentic in several ways. Some students did understand the game as a complex dynamic system. | PDA Screen | Wi-Fi signal strength | | PDA walkie-talkie | Outbreak @ the institute is their AR name | On Location Learning | | Presentation, collaboration inquiry, role play | Incorporating the affordances of AR games and the dynamic models of participatory simulations make possible new kinds of authentic science inquiry experiences.. |
| (Karthikeyan, Mani, Balasubramaniyan, & Selvam, 2012a) | To use AR along with serious games to improve the medical training process and user experience | Experiment questionnaire | | Not clear | The use of augmented reality in serious games will improve the learning process and also allows the user to interact with the game environment freely. | Not clear | EGM and GSR sensors | | Not clear | ARToolKit humanist serious games | None | | Game play | The use of augmented reality in serious games will improve the learning process. |
